# State-of-art neuroanatomical target analysis of high-definition and conventional tDCS montages used for migraine and pain control

**DOI:** 10.3389/fnana.2015.00089

**Published:** 2015-07-15

**Authors:** Alexandre F. DaSilva, Dennis Q. Truong, Marcos F. DosSantos, Rebecca L. Toback, Abhishek Datta, Marom Bikson

**Affiliations:** ^1^Headache and Orofacial Pain Effort (H.O.P.E.), Department of Biologic and Materials Sciences and Michigan Center for Oral Health Research (MCOHR), School of Dentistry, University of MichiganAnn Arbor, MI, USA; ^2^Department of Biomedical Engineering, The City College of New YorkNew York, NY, USA; ^3^Campus Macaé, Universidade Federal do Rio de Janeiro (UFRJ)Rio de Janeiro, Rio de Janeiro, Brasil; ^4^Soterix Medical, Inc.New York, NY, USA

**Keywords:** neuromodulation, finite-element modeling, transcranial direct current stimulation, pain, HD-tDCS

## Abstract

Although transcranial direct current stimulation (tDCS) studies promise to modulate cortical regions associated with pain, the electric current produced usually spreads beyond the area of the electrodes’ placement. Using a forward-model analysis, this study compared the neuroanatomic location and strength of the predicted electric current peaks, at cortical and subcortical levels, induced by conventional and High-Definition-tDCS (HD-tDCS) montages developed for migraine and other chronic pain disorders. The electrodes were positioned in accordance with the 10–20 or 10–10 electroencephalogram (EEG) landmarks: motor cortex-supraorbital (M1-SO, anode and cathode over C3 and Fp2, respectively), dorsolateral prefrontal cortex (PFC) bilateral (DLPFC, anode over F3, cathode over F4), vertex-occipital cortex (anode over Cz and cathode over Oz), HD-tDCS 4 × 1 (one anode on C3, and four cathodes over Cz, F3, T7, and P3) and HD-tDCS 2 × 2 (two anodes over C3/C5 and two cathodes over FC3/FC5). M1-SO produced a large current flow in the PFC. Peaks of current flow also occurred in deeper brain structures, such as the cingulate cortex, insula, thalamus and brainstem. The same structures received significant amount of current with Cz-Oz and DLPFC tDCS. However, there were differences in the current flow to outer cortical regions. The visual cortex, cingulate and thalamus received the majority of the current flow with the Cz-Oz, while the anterior parts of the superior and middle frontal gyri displayed an intense amount of current with DLPFC montage. HD-tDCS montages enhanced the focality, producing peaks of current in subcortical areas at negligible levels. This study provides novel information regarding the neuroanatomical distribution and strength of the electric current using several tDCS montages applied for migraine and pain control. Such information may help clinicians and researchers in deciding the most appropriate tDCS montage to treat each pain disorder.

## Introduction

Transcranial direct current stimulation (tDCS) continues to be investigated as a therapeutic tool for alleviating symptoms of patients with a large array of neurologic disorders. It has been described as a reliable and well-tolerated brain stimulation technique (Nitsche et al., 2003a,b; Iyer et al., 2005) where a constant low-amperage (usually 1–2 mA) electric current is delivered to the cortex via surface electrodes positioned over the scalp (Tremblay et al., 2014). Since the introduction of this technology in its current form, positive results have been reported in psychiatry (Martin et al., 2011, 2013; Blumberger et al., 2012; Demirtas-Tatlidede et al., 2013; Wolkenstein and Plewnia, 2013) and neurorehabilitation (Webster et al., 2006; Lindenberg et al., 2012). In addition, several studies have explored the contribution of tDCS in the treatment of numerous pain disorders, including fibromyalgia (Fregni et al., 2006b; Valle et al., 2009; Mendonca et al., 2011; Riberto et al., 2011; Villamar et al., 2013b), pain due to traumatic spinal cord injury (Fregni et al., 2006a; Soler et al., 2010; Jensen et al., 2013; Wrigley et al., 2013), chronic pelvic pain (Fenton et al., 2009), refractory orofacial pain (Antal and Paulus, 2011), postherpetic neuralgia (DosSantos et al., 2012), painful diabetic polyneuropathy (Kim et al., 2013), chronic neuropathic pain following burn injury (Portilla et al., 2013), neurogenic pain (Boggio et al., 2009a), trigeminal neuralgia (Hagenacker et al., 2014), low back pain (Schabrun et al., 2014) and migraine (Antal et al., 2011; DaSilva et al., 2012; Viganò et al., 2013). Moreover, according to a recent meta-analysis there is scientific evidence that anodal tDCS decreases pain levels in patients and increases sensory/pain thresholds in healthy individuals (Vaseghi et al., 2014). Table 1 summarizes the results of clinical studies that investigated the effects of tDCS for pain control. While the mechanisms by which tDCS modulates CNS activity are not fully understood, there is evidence that tDCS can modulate endogenous pain networks by affecting mu-opioid and glutamate/GABA neurotransmission, resulting in functional/structural neuroplasticity (DosSantos et al., 2012, 2014; Foerster et al., 2015). In addition, tDCS effects could be associated with thalamic inhibition mediated by cortico-thalamic fibers (Zaghi et al., 2009).

**Table 1:** **Summary of the studies found in the current literature investigating the effects of tDCS in different pain disorders**.

Trial	Stimulation	Montage	Inclusion	Sample	Results	Reference
Five sessions	Anodal or sham	HD-tDCS 2 × 2 H.O.P.E. Montage	Chronic TMD	24 patients	Significant pain relief above 50% in the VAS scale at four week follow-up; increased pain-free mouth opening at one week follow-up; and improvement of pain area, intensity and their sum measures contralateral to M1 stimulation during week of treatment. No changes in emotional values were shown between sham and activegroup.	Donnell et al. (2015)
28 sessions	Anodal or sham	M1-SO	Trigeminal neuralgia	10 patients	Anodal stimulation significantly reduced pain intensity (in a verbal rating scale), but not frequency of attacks.	Hagenacker et al. (2014)
Four sessions	Anodal or sham combined with active or sham peripheral electrical stimulation	M1-SO	Chronic recurrent low back pain	16 patients	tDCS combined with peripheral electrical stimulation caused significant pain reduction, with more pronounced results in subjects with greater central sensitization. However, when applied separately, none of the methods produced significant results.	Schabrun et al. (2014)
Single sessions	Anodal or sham	M1-SO	Chronic pain due to spinal cord injury	30 patients	Although the effect size of tDCS was 1.6 times as large as of sham, it was not enough to produce statistically significant differences.	Jensen et al. (2013)
Five sessions	Anodal or sham	M1-SO DLPFC-SO	Painful diabetic polyneuropathy	60 patients	Patients that received M1-SO stimulation displayed more significant reductions of pain, measured by a visual analog scale (VAS) and higher increases of pressure pain thresholds (PPT) when compared to DLPFC-SO and sham. The pain reduction lasted for2–4 weeks.	Kim et al. (2013)
Two sessions	Anodal and sham	M1-SO	Chronic neuropathic pain following burn injury	3 patients	No changes in the clinical outcomes analyzed.	Portilla et al. (2013)
Ten sessions	Anodal and sham	M1-SO	Neuropathic pain due to thoracic spinal cord injury	10 patients	Neither active nor sham tDCS resulted in significant pain relief, assessed by pain intensity (numerical rating scale and verbal rating scale) and unpleasantness.	Wrigley et al. (2013)
16 sessions	Anodal	Visual cortex, near Oz-chin	Episodic migraine without aura	13 patients	Anodal stimulation significantly decreased the number of days with migraine, frequency and duration of migraine attacks as well as acute medication intake.	Viganò et al. (2013)
Single sessions	Anodal, cathodal and sham	HD-tDCS 4 × 1	Fibromyalgia	18 patients	Pain reduction immediately after cathodal and evident 30 min after anodal and cathodal. Increase in mechanical pain threshold, bilaterally, after anodal stimulation.	Villamar et al. (2013b)
Ten sessions	Anodal or sham	M1-SO	Chronic migraine	13 patients	Positive but delayed analgesic effects: significant decrease of pain intensity and length of migraine episodes.	DaSilva et al. (2012)
Ten sessions	Cathodal or sham	Oz-Cz	Chronic and episodic migraine	30 patients	Significant reduction in the duration of attacks and pain intensity, but not in the frequency of attacks.	Antal et al. (2011)
Single sessions	Anodal, cathodal or Sham	Cathodal-M1 Cathodal-SO Anodal-M1 Anodal-SO Sham + Extracephalic electrode	Fibromyalgia	30 patients	Significant pain improvement, measured by a visual numerical scale (VNS) with cathodal-SO and anodal-SO. A trend to a similar effect in PPT with anodal-SO.	Mendonca et al. (2011)
Ten sessions	Anodal or sham+ Multidisciplinary rehabilitation program	M1-SO	Fibromyalgia	23 patients	Significant greater decreases of SF-36 pain domain scores and trend to greater improvement in the Fibromyalgia Impact Questionnaire (FIQ) scores in patients that received active tDCS.	Riberto et al. (2011)
Five sessions	Anodal or sham	M1-SO	Fibromyalgia, trigeminal neuralgia, poststroke pain syndrome and back pain	23 patients	Anodal tDCS resulted in a more pronounced reduction of pain (VAS), when compared to sham stimulation. The effects lasted for 3–4 weeks.	Antal et al. (2010)
Ten sessions	Anodal or sham combined with walking visual illusion or control illusion	M1-SO	Neuropathic pain due to spinal cord injury	39 patients	The combined intervention (tDCS + visual illusion) showed better and longer lasting effects on the overall severity of neuropathic pain and pain subtypes than the single interventions.	Soler et al. (2010)
Single sessions	Anodal or sham plus active or sham TENS	M1-SO	Neurogenic pain of the arms	8 patients	Significant pain reduction after tDCS and tDCS/TENS but not after sham tDCS. tDCS/TENS produced better results than tDCS alone.	Boggio et al. (2009a)
Four sessions	Anodal and sham	M1-SO	Chronic pelvic pain	7 patients	Significant decrease in pain, disability and traumatic stress scores after active tDCS.	Fenton et al. (2009)
Ten sessions	Anodal or sham	M1-SO DLPFC-SO	Fibromyalgia	41 patients	Both montages produced beneficial effects such as improvements of pain (measured by VAS) and quality of life (measured by FIQ). However, only M1-SO produced long-lasting clinical effects.	Valle et al. (2009)
Five sessions	Anodal or sham	M1-SO	Central pain due to traumatic spinal cord traumatic spinal cord injury	17 patients	Significant pain decrease after anodal stimulation, but not after sham stimulation. Such results were not confounded by changes in depression or anxiety. Lack of cognitive changes.	Fregni et al. (2006a)
Five sessions	Anodal or sham	M1-SO DLPFC-SO	Fibromyalgia	32 patients	Greater pain reduction after anodal M1 stimulation, when compared to sham and anodal DLPFC stimulation. The effected produced by M1 stimulation lasted for 3 weeks after the end of the treatment.	Fregni et al. (2006b)

The effects produced by tDCS are hypothesized to derive from neuronal membrane polarization, which is determined by the electric field generated in a given brain region. Unlike other methods of supra-threshold brain stimulation, such as deep brain stimulation (DBS) or transcranial magnetic stimulation (TMS), tDCS produces small sub-threshold electric fields (e.g., <1 V/m produced by tDCS vs. 100 V/m produced by other modalities) (Dmochowski et al., 2011). Thus, the degree of brain modulation by tDCS is presumed to monotonically reflect local electric fields. Moreover, while invasive methods (e.g., motor cortex stimulation, MCS) involve directly implanting the electrodes in cortical and/or subcortical structures, in non-invasive approaches (e.g., tDCS) the electric field is not restricted to the target region; instead, it spreads over neighboring cortical and even subcortical regions according to the configuration or montage applied (Bikson et al., 2013). The tDCS montage, as well as other factors such as brain structure (including white and gray matter and regional boundaries) and thickness of the cerebrospinal fluid (CSF) and the skull, determine the electric field generated in each brain region and hence the propensity to modulate regional function (Opitz et al., 2015). Our analysis focuses on the overlap between montage-specific brain current flow and nodes in the pain network. Based on the results of a previous study, we expect to find peaks of electrical current flow in outer cortical areas, including the prefrontal cortex (PFC), primary somatosensory cortex (S1), primary motor cortex (M1) and visual cortex, as well as the insula, cingulate cortex, thalamic nuclei and brainstem. Furthermore, we hypothesize that high-definition (HD-tDCS™) montages will produce more focal effects when compared to conventional tDCS montages.

In order to optimize the distribution of the electric current delivered to the central CNS, computational models have been developed, which predict the patterns of the electric current flow (Datta et al., 2011). In this study, we used high-resolution computational models to investigate the neuroanatomical distribution and peak strength of the current flow across regions of interest in pain control during five different types of tDCS: three conventional and two HD-tDCS montages. The electrodes were positioned following the 10–20 or 10–10 electroencephalogram (EEG) landmarks, typically adopted in tDCS administration. In the conventional setup, two large electrodes (5 × 7), corresponding to the anode (positive pole) and cathode (negative pole), were combined to produce three basic configurations: M1-SO (motor cortex-supraorbital: anode positioned over C3 and cathode positioned over Fp2; DaSilva et al., 2011), DLPFC (dorsolateral PFC bilateral: anode over F3, cathode over F4) and Cz-Oz (vertex-occipital cortex: anode and cathode over Cz and Oz, respectively). In addition, two HD montages were applied using ring electrodes, with the purpose of increasing spatial tDCS focality: HD-tDCS 4 × 1 (one anode centered on C3, surrounded by four cathodes over Cz, F3, T7, and P3) and HD-tDCS 2 × 2 (two anodes over C3 and C5 and two cathodes over FC3 and FC5) (Datta et al., 2009; Villamar et al., 2013a).

## Materials and Methods

Brain current flow (electric fields) through cortical and subcortical structures was evaluated based on previous studies and defining putative Brodmann Areas (BA; Wiegell et al., 2003; Afif and Mertens, 2010; Mai and Paxinos, 2012). To compare current flow patterns across the five different electrode montages, we developed an individualized finite element (FE) head model (Huang et al., 2013). Tissues and other materials of varying properties, namely conductivity, were identified and segmented from a high-resolution 1 mm^3^ T1 MRI of a neurologically normal adult male. Automated segmentation algorithms (SPM8, Wellcome Trust Center for Neuroimaging, London, UK) with customized tissue probability maps and filters specific for FE modeling (Huang et al., 2013) created the initial tissue geometry and ensured continuity in CSF. Additional manual segmentation using a digital pen and tablet resolved finer detail throughout the head including cortical folding and deeper brain structures. Geometric models of sponge electrodes (5 × 7 cm) and HD electrodes (12 mm diameter) were created as computer-aided design (CAD) files and incorporated into the image volume (Truong et al., 2012). The resulting volumetric image data was then converted into meshes using adaptive voxel-based meshing algorithms (Simpleware Ltd, Exeter, UK). The meshes were imported into an FE package (COMSOL Multiphysics 4.3, COMSOL, Inc., Burlington, MA, USA) where electrostatic physics were applied (Datta et al., 2009). The Laplace equation with purely conductive properties was solved for electric field (Miranda et al., 2006; Wagner et al., 2007; Datta et al., 2009) and the following isotropic electrical conductivities were assigned (S/m): skin: 0.465, fat: 0.025, skull: 0.01, CSF: 1.65, gray matter: 0.276, white matter: 0.126, air: 10^−15^, saline-soaked sponge or conductive gel: 1.4, electrode: 5.99 × 10^7^. Current density corresponding to 1 mA total current was applied at the anode(s), as is typically used for tDCS, and ground applied to the cathode(s). All other surface boundaries, primarily skin in contact with air, were considered electrically insulated. The entire model workflow preserved precision beginning from the 1 mm^3^ resolution MRI to the induced cortical electric field maps (Bikson and Datta, 2012). Stimulation electrodes for all the montages were positioned in accordance with the 10–20 or 10–10 EEG landmarks as is used in typical tDCS administration.

Five montages were simulated:

- *M1-SO*: 5 × 7 cm sponges with anode positioned vertically over 10–20 location C3 and cathode positioned horizontally on the contralateral-supraorbital, approximately over 10–20 location Fp2.
- *DLPFC (F3-F4)*: 5 × 7 cm sponges with anode and cathode positioned vertically over 10–20 locations F3 and F4 respectively.
- *Cz-Oz*: 5 × 7 cm sponges with cathode positioned horizontally over 10–20 location Oz. Anode is centered on the vertex, Cz, with the length of the pad parallel to the line from ear to ear.
- *HD-tDCS 4 × 1*: 12 mm diameter disk electrodes with the anode centered on 10–20 location C3. Cathodes (4) surround the anode 90° apart at a “4 × 1 ring” radius of approximately 75 mm. Explicit cathode locations were 10–10 locations Cz, F3, T7, and P3.
- *H.O.P.E. HD-tDCS 2 × 2* (Donnell et al., 2015): 12 mm diameter disk electrodes with two anodes and two cathodes positioned posterior to anterior across the face/head region of M1. Explicit 10–10 anode locations are C3 and C5. Cathode locations are FC3 and FC5.

## Results

**Figure 1:**
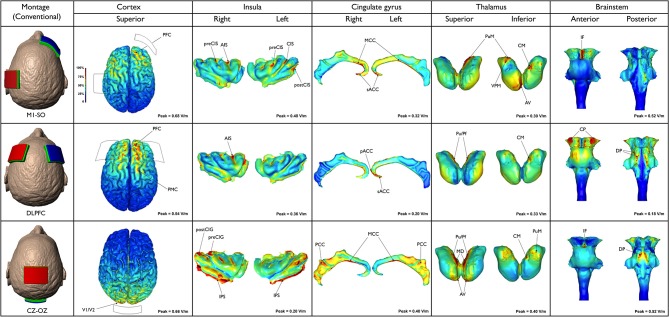
**Three different conventional tDCS montages are illustrated (first column, from the top to the bottom): M1-SO motor (cortex-supraorbital), DLPFC (dorsolateral prefrontal cortex bilateral) and Cz-Oz (vertex-occipital cortex)**. Electric field maps generated in outer cortical regions and inner structures (insula, cingulate gyrus, thalamus and brainstem) are illustrated in the next five columns (from the left to the right).

**Figure 2:**
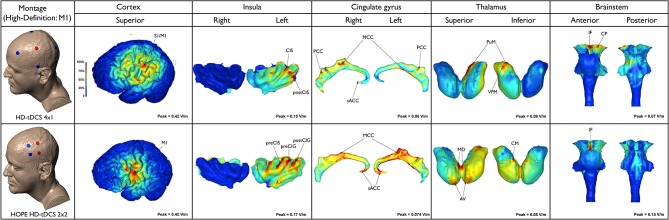
**Electric field maps produced by two methods of high-definition (HD)-tDCS: HD-tDCS 4 × 1 and H.O.P.E. HD-tDCS 2 × 2 (first column from the top to the bottom) in outer (second column) and inner structures (third to sixth, from the left to the right)**.

The neuroanatomical current distribution and the strength of the predicted electric current peaks related to conventional and High-Definition-tDCS (HD-tDCS) montages are illustrated in Figures 1 and 2, respectively.

### M1-SO

The frontal lobe received a larger amount of current when compared to other cortical structures. Nonetheless, the current spread over several regions. Peaks of current density and in turn electric field (0.68 V/m) occurred in the PFC, including parts of the superior, middle and inferior frontal gyri, bilaterally. In addition, the current flowed to the premotor cortex (PMC), putative BA 6, and precentral gyrus (primary motor cortex—M1, putative BA 4) particularly on the left side. Peaks of current were also found in the anterior and posterior insula on both sides. Interestingly, the highest insular current density occurred in depth of the anterior insular sulcus (AIS), precentral (preCIS), central (CIS) and postcentral (postCIS) insular sulci, especially on the left side. In the cingulate cortex the current flow was more discrete, extending from the subgenual anterior cingulate cortex (sACC) to the midcingulate cortex (MCC) bilaterally, though with higher intensities on the right side. According to the results of the current study, the thalamus is also directly stimulated in this type of montage. Peaks of current flow were found in the medial pulvinar (PuM), ventral posteromedial (VPM) and anteroventral (AV) nuclei as well as the centromedian (CM) nucleus on the right side. In the brainstem peaks were concentrated in the midbrain, specifically along the interpeduncular fossa. Moreover, few peaks were detected on the left cerebral peduncle. Regions presenting lower densities of current flow were also found in the dorsal pons and medulla.

### DLPFC (F3–F4)

The peak magnitude of the electric field (0.54 V/m) occurred in the right PFC, mainly in anterior parts of the superior and middle frontal gyri. On the left hemisphere the current was more limited to the anterior part of the superior frontal gyrus. Part of the electric current also spread to the right precentral PMC. A relatively intense electric field (>0.36 V/m) was predicted in the right AIS, while scarce electric fields were predicted at the sACC and pregenual anterior cingulate cortex (pACC). Few thalamic clusters were predicted when DLPFC montage was applied, mainly projected to the right and left PuM and right CM. Electric field peaks (0.18 V/m) were localized in the most lateral aspects of the right and left cerebral peduncles. Current density, however, was relatively low (0.0227 A/m^2^) due to the lower conductivity used in modeling brainstem (*σ* = 0.126 S/m vs. 0.276 S/m for cortex, where *J* = E/σ). Both the ventral and dorsal parts of the pons exhibited peaks of current density. Increased current densities were detected in the superior and inferior cerebellar peduncle, extending to a tiny area in the most cranial part of the medulla, including the olives.

### Cz-Oz

Using this montage, the current flowed mainly to the parietal and occipital lobes with the maximum electric field (0.66 V/m) occurring in the primary visual cortex V1 (putative BA 17) and secondary visual area V2 (putative BA 18). Large areas of intense current flow were found in the inferior peri-insular sulcus (IPS) and posterior insula, bilaterally. Peaks were also detected in the right precentral (preCIG) and postcentral (postCIG) insular gyri. Moreover, peaks of electric field (0.40 V/m) were predicted at several parts of the cingulate cortex, bilaterally—more so than in any other montage tested. Peaks were mainly predicted in the MCC and posterior cingulate cortex (PCC) on both sides. Thalamic clusters were located in the following nuclei: pulvinar (Pu), parafascicular (Pf), mediodorsal (MD) and AV, bilaterally, as well as right CM and PuM. In the brainstem, a cluster of increased current flow occurred in the interpeduncular fossa, while larger areas of great current flow were found in the dorsal pons, medially to the superior cerebellar peduncle. Lower current densities also occurred in the midbrain tectum.

### 4 × 1 HD-tDCS M1

As expected, this model limited the anatomical distribution of the electric field to within the ring perimeter. The current flowed to only a few areas in the outer cortical regions and remained exclusively on the side of the stimulation. Peaks of current were located in the depth of the central sulcus and adjoining structures, including the precentral gyrus (M1) and postcentral gyrus (primary somatosensory cortex—S1, putative BA 3, 1, 2). The latter had the highest predicted electric field (0.42 V/m) among the superficial cortical structures. Increased electric current also occurred in the inferior parietal lobule (supramarginal area, putative BA 40) and postcentral sulcus. Clusters of electric activity (0.15 V/m) were also found in the anterior and posterior parts of the left insula (e.g., CIS and postCIS). In the cingulate cortex, the most intense current flow occurred in the right/left MCC, PCC, as well as the right sACC. The left PuM and VPM received the majority of the thalamic current flow. In the brainstem, the electric current flowed to the interpeduncular fossa and left cerebral peduncle. Small clusters occurred on the left dorsal pons.

HD-tDCS can be delivered using many different positions and number of electrodes, with each montage optimized for a specific clinical or experimental objective. With 4 × 1 montage for instance, a wider ring leads to a wider, intense, and deeper region of induced cortical current flow, while decreasing the ring diameter leads to increased focality at the cost of increased current shunting across the scalp (without crossing into the cortex). Therefore, the depth, extent, and maximal intensity of induced cortical current flow can be titrated by simply changing ring diameter (Edwards et al., 2013).

### 2 × 2 HD-tDCS M1

The peak electric field (0.42 V/m) was predicted in the region between the anodes and cathodes. The current flow was even more localized when compared to the 4 × 1 montage, in lieu of the fact that the 4 × 1 montage employed a 15 cm diameter ring. Owing to reduced anode-cathode electrode separation (4 cm), cortical current flow was restricted to a reduced area resulting in increased focality for the 2 × 2 montage. The current flowed to the ipsilateral M1 and PMC. Current density peaks (0.047 A/m^2^) also occurred along the anterior and posterior left insula, particularly affecting the preCIS, preCIG and postCIG. Current flow occurred in the sACC and MCC, bilaterally. Thalamic clusters were detected in the AV and MD nuclei, on both sides and left CM. Furthermore, peaks of current occurred in the MD and CM/Pf nuclei, bilaterally. However, these were clearly more intense on the right side. In this montage the brainstem peak was very well localized and concentrated in the interpeduncular fossa. Noteworthy, the peaks of current flow found in the cingulate, thalamus and brainstem with HD-tDCS (4 × 1 or 2 × 2) were much lower, even negligible, when compared to those produced by conventional tDCS (M1-SO, F3-F4 or Cz-Oz).

## Discussion

Despite the vast number of studies exploring and challenging the clinical outcomes produced by tDCS (O’Connell et al., 2010, 2014), its impact in the CNS functioning has not been completely established. Hence, with recent evidence that tDCS can directly modify the activity of deeper brain structures through the diffusion of the current from extracranial electrodes (DaSilva et al., 2012), modeling studies have gained considerable importance (Edwards et al., 2013; Truong et al., 2013; Villamar et al., 2013b; Kuo et al., 2014; Ruffini et al., 2014). Previously in a migraine trial, researchers attempted to characterize the tDCS-induced electric current flow to the entire cortical surface and deeper brain structures, and found precisely defined peaks in four brain regions: insula, cingulate, thalamus and brainstem. In that proof of concept study (DaSilva et al., 2012), all areas analyzed contained significant peaks of electric current in subregions related to pain perception and analgesia. However, only the M1-SO montage used in the migraine trial was investigated. Thus, the neuroanatomical patterns of the current flow in other pain modulatory tDCS montages still needed to be explored and compared.

This study aimed to investigate the neuroanatomical distribution of electric current flow in five recently reported tDCS montages used for migraine and/or pain modulation. We hypothesized that peaks of current flow would be observed in outer cortical regions (e.g., PFC, S1, M1, V1 and V2) as well as deeper structures (e.g., insula, cingulate cortex, thalamic nuclei and brainstem), depending on the selected tDCS montage. As expected, there were significant differences in the neuroanatomical maps of current flow generated by each tDCS montage (Figures 1, 2). Confirming the primary hypothesis, HD-tDCS resulted in more focused cortical effects when compared to conventional montages. This finding was particularly evident with HD-TDCS H.O.P.E. 2 × 2 montage (Donnell et al., 2015). The current flow with the 2 × 2 montage was more concentrated than the 4 × 1 montage, as the 2 × 2 montage emulates the effects of implanted motor cortex stimulation by employing a much smaller anode-cathode electrode separation and postero-anterior electric current direction. In both HD-tDCS montages the current peaked in the ipsilateral precentral gyrus (M1) and postcentral gyrus (S1). However, current peaks for 2 × 2 and 4 × 1 HD-tDCS montages became negligible in (sub) cortical regions outside their small HD-tDCS electrodes’ ring, including the thalamus, ACC and insula, when compared to conventional montages. This focused targeting corroborates with the clinical outcome of a recent 4 × 1 M1 HD-tDCS study with fibromyalgia patients where a single session led to significant reduction in overall perceived pain (Villamar et al., 2013b). In another study, daily sessions of 2 × 2 M1 HD-tDCS in chronic temporomandibular disorders (TMD) patients induced significant pain relief—greater than 50% decrease in visual analog scale (VAS) pain ratings—at 4 week follow-up and pain-free mouth opening at 1 week follow-up (Donnell et al., 2015). Furthermore, during the treatment week there was significant improvement in the TMD pain area, intensity and their sum measures in the side contralateral to the M1 stimulation, but not in the ipsilateral side. Interestingly, there were no changes in emotional values between active and placebo groups, which indicates that M1 HD-tDCS montages, at least the 2 × 2, are precise in modulating sensorimotor functions.

Remarkably, in the present study M1-SO and DLPFC montages produced significant peaks of electric current in the PFC, a region intrinsically related to cognitive and emotional functions (Gusnard et al., 2001; Phelps et al., 2004; Tabibnia et al., 2014; Haas et al., 2015; Kong et al., 2015; Nakagawa et al., 2015) and thus important for the modulation of the emotional dimension of pain (Lorenz et al., 2002; Porro et al., 2002; Apkarian et al., 2004; Kuchinad et al., 2007; DaSilva et al., 2008; Boggio et al., 2009b; Metz et al., 2009; Jin et al., 2013; Baliki et al., 2014; Bogdanov et al., 2015). Based on the results of the current study, it is possible to speculate that not only DLPFC tDCS but also M1-SO produces its clinical effects through direct PFC modulation. However, further studies will be necessary to scrutinize this hypothesis.

Furthermore, M1-SO was the conventional montage with more significant effects in the insula, with peaks of current found in both the posterior and anterior insula, while the DLPFC montage resulted in large amounts of electric current in the anterior insula only. Several studies have established the major role of insula in pain processing (Ostrowsky et al., 2002; Craig, 2003; Apkarian et al., 2005; Brooks et al., 2005; Brooks and Tracey, 2007; Henderson et al., 2007; Frot et al., 2014), and structural and functional changes in the insular cortex have been demonstrated in numerous pain conditions, including trigeminal neuropathic pain (DaSilva et al., 2008; Moisset et al., 2011), cluster headache (Sprenger et al., 2007), and migraine (Kim et al., 2008; Schmidt-Wilcke et al., 2008; Valfre et al., 2008; Prescot et al., 2009; Coppola et al., 2014). However, according to neuroimaging studies, there are functional differences between the anterior and posterior insula. While the anterior insula is involved in emotional functions, the posterior insula is more related to visceral symptoms (Dupont et al., 2003). In addition, the nociceptive input is first processed at the posterior insula, which is likely related to the interpretation of the anatomical location and intensity of the stimulus, and then at the anterior insula, mainly involved in emotional reactions (Frot et al., 2014). Other studies revealed that clinical pain is more rostrally located in the anterior insula than acute experimental pain induced in healthy subjects (Schweinhardt et al., 2006; Brooks and Tracey, 2007; Schweinhardt and Bushnell, 2010). Therefore, while both montages (M1-SO and DLPFC) are likely suited to modulate high emotional involvement commonly seen in chronic pain patients, M1-SO should be preferentially used when aiming to treat visceral pain.

Regarding the activation of medial neuroanatomical regions, Cz-Oz was the conventional montage with more significant effects in the MCC and PCC, while the M1-SO and DLPFC montages preferentially reached the ACC and the anterior MCC. Previous neuroimaging studies reported changes in the cingulate cortex of migraine patients, mostly in the ACC and MCC (Weiller et al., 1995; Kim et al., 2008; Schmidt-Wilcke et al., 2008; Valfre et al., 2008; May, 2009) but also in the PCC (Kim et al., 2008). Furthermore, the results of a recent investigation suggest that changes in metabolite levels occur in the PCC of patients with chronic pain, including migraine (Fayed et al., 2014). Thus, the decrease of pain found in migraine patients after M1-SO tDCS (DaSilva et al., 2012) could be explained not only by the stimulation of the cortical regions beneath the electrode and indirect activation of more remote regions, but also by a direct effect in the ACC and/or anterior parts of the MCC. On the other hand, the positive results reported with Cz-Oz in migraine (Antal et al., 2011; Siniatchkin et al., 2012; Viganò et al., 2013) could be linked to the direct effects of tDCS in the posterior parts of the MCC or even PCC. Noteworthy, the presence of highly conductive intracerebral artery (which is assigned the conductivity of CSF) directly under the CZ pad provides a preferential conduit for currents to travel into medial brain regions. This likely explains the increased current flow in the posterior cingulate, thalamic nuclei and brainstem when using the Cz-Oz montage. Thus, Cz-Oz could be effective in migraine, not merely because of its action at the occipital (visual) cortex, but also due to its direct influence in medial structures related to pain processing and analgesia, such as the cingulate and thalamus. The thalamus is an important area for pain (Derbyshire et al., 1994; Hsieh et al., 1995; Iadarola et al., 1995; Casey et al., 1996; Vogt et al., 1996; May et al., 1998; Peyron et al., 1998; Petrovic et al., 1999; Rocca et al., 2010) as well as placebo effect (Wager et al., 2007; Zubieta and Stohler, 2009), and the pulvinar has been recently linked to migraine mechanisms (Moulton et al., 2011; Maleki et al., 2012; Schwedt et al., 2013). However, while thalamic current flow was detected in all montages (particularly in the pulvinar), robust levels of electric current were only detected with conventional montages compared to lower levels that occurred with HD-TDCS.

Despite the valuable information provided by modeling studies, it is still not possible to precisely define the extent to which the strength of the electric current correlates to the clinical effects reported with tDCS, as the mechanisms whereby nervous tissue is stimulated by this method are not completely understood. It has been hypothesized that tDCS effects derive from neuronal membrane polarization, which is determined by the electric field generated (Dmochowski et al., 2011). Moreover, evidence from TMS studies indicates that nervous system stimulation takes place at the electrical field peaks (Amassian et al., 1994; Wassermann et al., 1996; Krings et al., 1997; Boroojerdi et al., 1999; Miranda et al., 2003). Nonetheless, further studies are necessary to establish the mechanisms by which tDCS acts at the central nervous system.

## Conclusion

The present study provides information on the electric current flow generated by different conventional and HD-tDCS montages applied for migraine/pain control. Five montages were analyzed (M1-SO, DLPFC, Cz-Oz, 4 × 1 HD-tDCS and 2 × 2 HD-tDCS) and all produced significant results. Nevertheless, an increased focality occurred with HD-tDCS. As this study demonstrates, the neuroanatomical approach, based on computational models, is crucial to define the neural networks directly modulated by each type of tDCS montage applied for pain investigation and relief. It is important to emphasize that the single subject models used for this study provide data on broad variations in current flow patterns that are expected to generalize. However, this information ultimately contributes to a broader comprehension of the neurophysiologic aspects as well as central pain mechanisms targeted by tDCS. In the future, the combination of brain-modeling analysis with the evaluation of specific functional and/or structural neuroplastic changes related to pain could contribute to identify the most appropriate tDCS montage to treat each chronic pain disorder.

## Author Contributions

AFD and MB mentored, conceived, designed, obtained funding and coordinated the study. They also drafted the manuscript. DQT and AD acquired the data, designed the study, analyzed the data and drafted the manuscript. MFD designed the study, analyzed the data, and drafted the manuscript. RLT contributed to the study and helped to draft the manuscript. All authors read and approved the current version of the manuscript.

## Conflict of Interest Statement

Dr. Datta and Dr. Bikson are co-founders of Soterix Medical. The other authors declare no conflicts of interest related to this study.

## Abbreviations

AIS: anterior insular sulcus
AV: anteroventral
BA: Brodmann area
CAD: computer-aided design
CIS: central insular sulcus
CM: centromedian
CP: cerebral peduncle
CZ: vertex
DLPFC: dorsolateral prefrontal cortex
DP: dorsal pons
EEG: electroencephalogram
HD: High-Definition
IF: interpeduncular fossa
IPS: inferior peri-insular sulcus
MCC: midcingulate Cortex
MD: mediodorsal
M1: primary motor cortex
OZ: occipital cortex
PFC: prefrontal cortex
pACC: pregenual anterior cingulate cortex
PCC: posterior cingulate cortex
Pf: parafascicular
PMC: premotor cortex
postCIS: postcentral insular sulcus
postCIG: postcentral insular gyrus
preCIG: precentral insular sulcus
preCIS: precentral insular sulcus
Pu: pulvinar
PuM: medial pulvinar
P3: left parietal cortex
sACC: subgenual anterior cingulate cortex
SO: supraorbital region
S1: somatosensory cortex
TMD: temporomandibular disorders
T7: left temporal cortex
VPM: ventral posteromedial
V1: primary visual cortex
V2: secondary visual cortex.
